# Metabolic response to burn injury: a comprehensive bibliometric study

**DOI:** 10.3389/fmed.2024.1451371

**Published:** 2025-01-03

**Authors:** Yixu Li, Yifan Liu, Sujie Xie, Yushu Zhu, Xinran Ding, Wei Zhang, Shuyuan Xian, Guosheng Wu, Hanlin Sun, Jiale Yan, Bingnan Lu, Yuntao Yao, Weijin Qian, Yuwei Lu, Yiting Yang, Dayuan Xu, Runzhi Huang, Shizhao Ji

**Affiliations:** ^1^Department of Burn Surgery, The First Affiliated Hospital of Naval Medical University, Shanghai, China; ^2^Research Unit of Key Techniques for Treatment of Burns and Combined Burns and Trauma Injury, Chinese Academy of Medical Sciences, Shanghai, China; ^3^Department of Urology, Xinhua Hospital Affiliated to Shanghai Jiao Tong University School of Medicine, Shanghai, China; ^4^Shanghai Jiao Tong University School of Medicine, Shanghai, China; ^5^Department of Gynecology, Shanghai First Maternity and Infant Hospital, Tongji University School of Medicine, Shanghai, China

**Keywords:** thermal injury, burn injury, metabolism, inflammatory, management

## Abstract

**Objective:**

Burns lead to systemic changes manifested by systemic disturbances in water-electrolyte balance and systemic metabolic and inflammatory responses. The hypermetabolic response after a burn injury relies on metabolic, hormonal, and inflammatory dysregulation mechanisms. This study aimed to provide a comprehensive bibliometric analysis of the burn metabolism research field, identifying key trends, influential contributors, and emerging research hotspots to inform future investigative efforts. Ultimately, we conducted an extensive review of the literature, synthesizing the findings to clarify the present understanding within our field of study.

**Methods:**

We obtained 8,823 scientific publications on burn injury and metabolism from the core Web of Science (WOS) database collection. In this work, biblioshiny was used to visualize and analyze the data, and VOSviewer was used to verify the results.

**Results:**

From a total of 8,823 publications, we found a general upward trend in annual publications and citation frequency. According to Bradford’s Law, 21 high-production journals were classified as core sources based on the number of publications, and the most productive journal was Burns. The most published countries and authors in this field were the United States and Herndon DN. The most local cited document in this field was the article titled “Catecholamines: Mediator of the Hypermetabolic Response to Thermal Injury” authored by Wilmore DW. The thematic map showed that studies on injury, thermal injury, and sepsis were relatively mature. In contrast, research on metabolism, stress, and responses, and research on mortality, resistance, and management were less well-developed but were essential for the field.

**Conclusion:**

Research on burns and metabolism is increasing. Based on the bibliometric analysis, our study summarized the complex interplay between burn-induced systemic metabolic alterations and inflammatory responses, emphasizing the significance of hypermetabolism and its management. The role of propranolol, insulin, oxandrolone, and nutritional interventions in modulating the hypermetabolic state was discussed. Additionally, our study underscored the challenges of managing sepsis and drug-resistant infections in burn patients as an important future area of research.

## 1 Introduction

Burn injuries constitute a profoundly impactful and pervasive global public health crisis, encompassing a spectrum of traumatic experiences resulting from the application of heat, radiation, electric current, or chemical agents to the skin or other bodily tissues, thereby inflicting severe damage (1). Thermal injuries are caused by excessive heat, typically from contact with steam or flames (2). Thanks to advancements in protective equipment and the strides made in science and medical technology, the incidence and mortality rates associated with burns have witnessed a general decline (3). However, the incidence of burns remains high in low- and middle-income areas, and the enduring suffering experienced by burn patients remains a poignant reality. The harmful effects of burns extend beyond the evident damage to the skin barrier, encompassing profound alterations in the body’s internal environment, precipitating an intense stress response (4).

Central to the pathophysiology of burn injuries is the persistence of hypermetabolism, notably characterized by hypercatabolism (5). The hypermetabolic response after a thermal injury is based on metabolic, hormonal, and inflammatory dysregulation mechanisms and is characterized by elevated glucose production, intensified lipolysis, robust protein catabolism, and a substantial surge in energy consumption (6). Such a hypermetabolic state leads to considerable energy expenditure and can impact multiple organ systems, presenting a persistent challenge in clinical management. Expertly navigating this metabolic upheaval requires vigilant clinical intervention and monitoring. Previous investigations posit that hypercatabolism is intricately linked to the stress response (7–9), thereby engendering a cascade of pathological changes, which is not conducive to the recovery of patients (6).

Bibliometrics is a multidisciplinary science amalgamating mathematics, statistics, and bibliography. It facilitates quantitative data analysis about publications (e.g., authors, countries, affiliations, keywords, citations) (10). By deploying bibliometrics, one can discern research hotspots within a specific field and prognosticate potential trends on the horizon. Despite its widespread application across various disciplines (11–15), bibliometrics has not yet been extensively employed to support investigations into burn metabolism. Therefore, we analyzed the scientific literature of studies on burn metabolism from the Web of Science (WOS) core database using bibliometrics to find research hotspots and summarize previous research findings to inform future research in the field of burn metabolism.

## 2 Method

### 2.1 Research strategy

The WOS database was searched via the search formula ((TS = scald burns) OR (TS = scald burn) OR (TS = burn injury) OR (TS = burn injuries) OR (TS = ambustion injury) OR (TS = ambustion injuries) OR (TS = scald injury) OR (TS = scald injuries) OR (TS = empyrosis) OR (TS = scalds) OR (TS = scalding) OR (TS = scalded)) AND ((TS = metabolism) OR (TS = metabolic)). Only reviews and articles were retrieved to ensure academic rigor and consistency of the findings. The relevant information of the remaining 8,823 publications was downloaded in TXT format, and the raw data can be found in Supplementary Digital Content 1. All searches and data extraction were performed on 10 July 2023 to avoid bias.

### 2.2 Data analysis

Bibliometrix (16), a software proficient in bibliometric analyses and programmed in R version 4.2.3, offers exceptional flexibility and utility. In this article, we imported all the results into Bibliometrix. We conducted an in-depth analysis of annual scientific articles, countries, affiliations, authors, documents, journals, and keywords in the context of burns and metabolism research. These indicators included citation frequency, multiple country publications (MCP), single country publications (SCP), total citations (TC), and number of documents. Besides, we also used local citations to show how often an author (or a document, or a journal) included in this collection was cited by the documents included in the collection, and global citations to measure the TC that a country/region has received from documents indexed in the WOS database (16). MCP ratio is calculated by dividing the number of multiple country publications by the total number of publications in that domain for the country to measure the extent of international collaboration of the country (17). To further evaluate the influence of authors and publications, we utilized the H-index, which provides a comprehensive measure of scientific output and impact (18). To identify the core sources in our field, we applied Bradford’s Law, a quantitative approach that describes the concentration and dispersion of professional papers across related journals. We utilized word cloud and tree map to highlight high-frequency keywords and used a co-occurrence network to demonstrate the frequency at which keywords appeared together, unveiling the relationships and connections between different research areas. We employed thematic maps and analyzed thematic evolution to explore the development of hot topics and potential research directions. These techniques helped us gain insights into emerging trends, identify research hotspots, and speculate on potential areas of future exploration. VOSviewer (version 1.6.19) is a computer program for bibliometric mapping (19). It was used for supplementary analysis and validation of results.

## 3 Result

### 3.1 Annual publication trends

**Figure 1:**
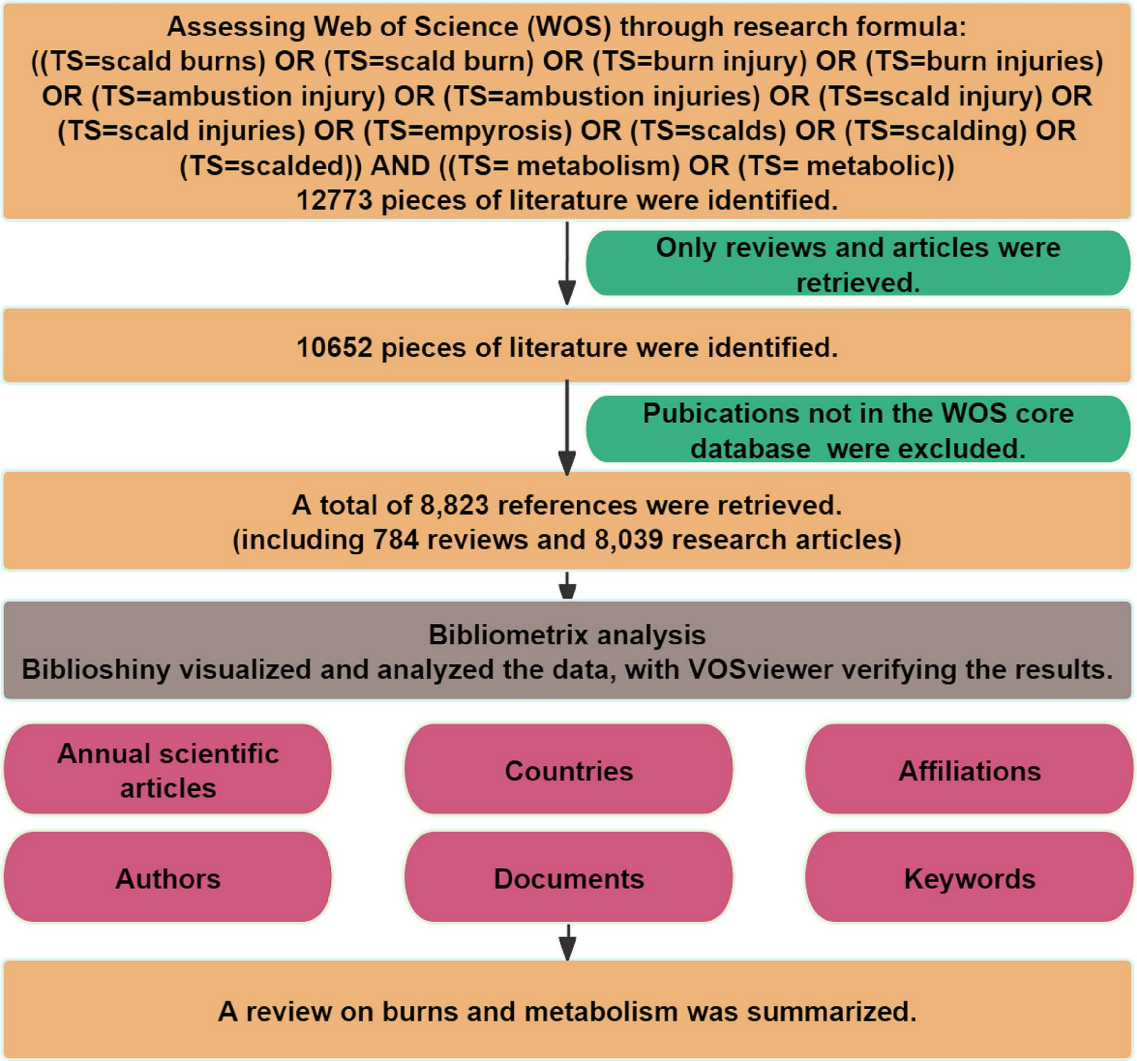
Analytic process of metabolism and burns research.

In our investigation, we utilized the WOS core database, focusing on reviews and articles. A total of 8,823 references were retrieved. The flowchart of our analysis is shown in Figure 1. Supplementary Figure S1 illustrated the annual publications and citation frequency. Since 1968, research at the intersection of metabolism and burns had entered a phase of rapid development. The annual number of publications and citation frequency had continuously risen, with a notable acceleration in citation frequency observed since 2017. This discernible trend underscored the increasing significance of burns and metabolism research in the scholarly landscape.

### 3.2 Most influential countries/regions and affiliations

**Figure 2:**
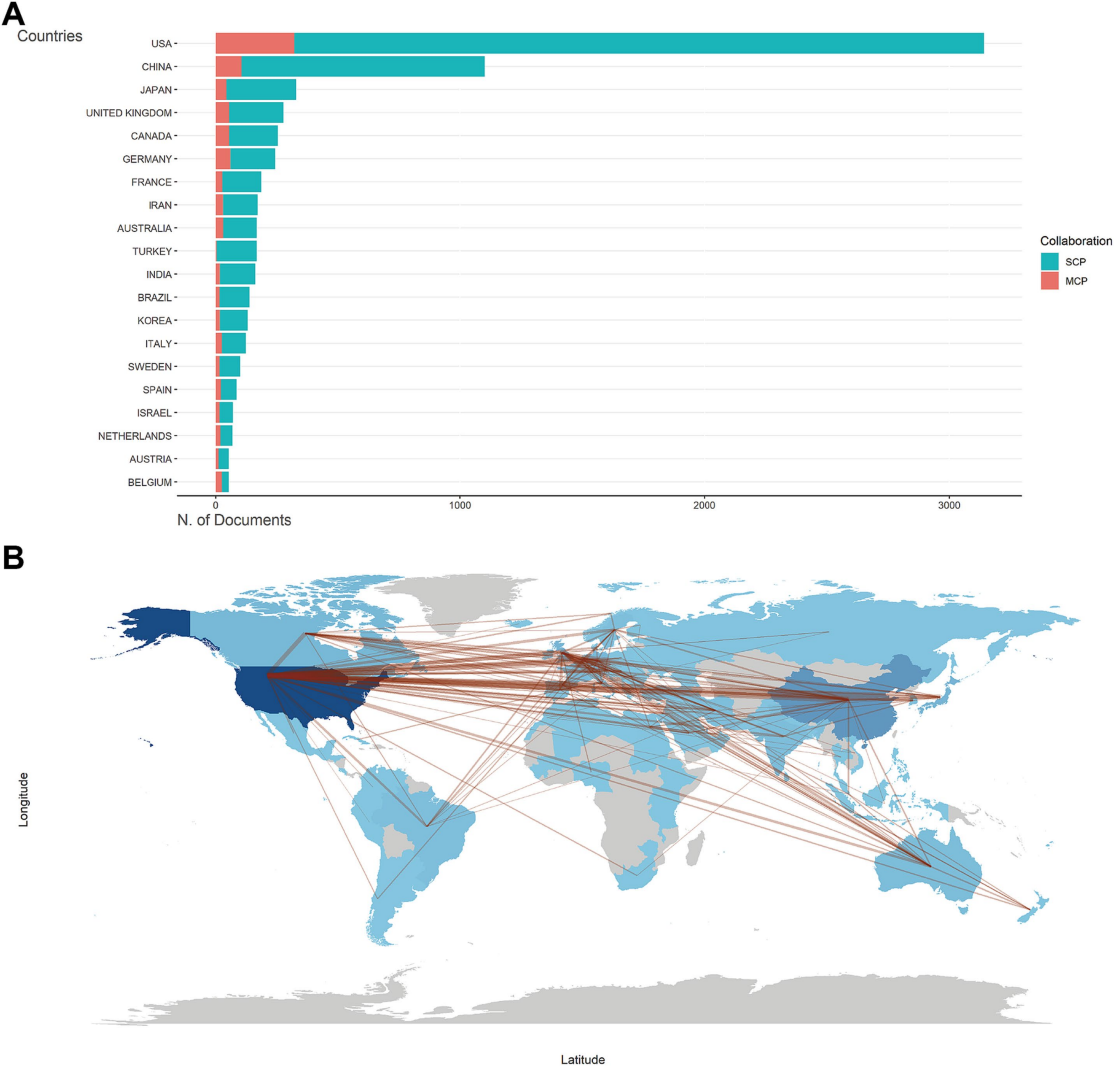
Countries/regions analysis. **(A)** Top 20 countries/regions of corresponding authors for metabolism and burns research. SCP, single-country publications. MCP, multiple-country publications. **(B)** Countries/regions production and collaboration world map for metabolism and burns research. The number of publications is shown in blue, with darker colors representing a larger number. The red lines between countries/regions refer to collaborations between them, and the thickness of the lines represents the strength of international collaborations.

Supplementary Table S1 listed the number of total citations, articles, SCP, and MCP for the top 20 most productive countries/regions. The United States was the foremost contributor and the most influential country in the field, with SCP and MCP dominating the top (Figure 2A). The collaboration world map (Figure 2B) illustrated that the United States served as the central connecting point, indicating its extensive collaborations with other publishing nations. Supplementary Figure S2A highlighted that Georgia, Nepal, and Singapore boasted the highest average citation of literature. This may be due to their collaboration with other countries, as we found that they had a higher MCP ratio and became the countries with the highest average citation rate of articles despite the low number of articles published (Supplementary Table S2). While the United States, China, and the United Kingdom were frequently cited (Supplementary Figure S2B), they did not hold the highest average citation. Supplementary Figure S2C showed an upward trend in publications of each country. The United States and China showed the fastest growth rates.

Among the affiliations contributing to the literature on burn metabolism, the University of Texas System, University of Texas Medical Branch Galveston, and Harvard University emerged as the top three institutions, as delineated in Supplementary Table S3. Affiliations’ production over time (Supplementary Figure S3) revealed a discernible uptrend in burn metabolism research across the institutions. Particularly noteworthy were the University of Texas Medical Branch Galveston and the University of Texas System, having experienced a significant surge in their publications in recent years.

### 3.3 Most productive authors and most cited reference

**Figure 3:**
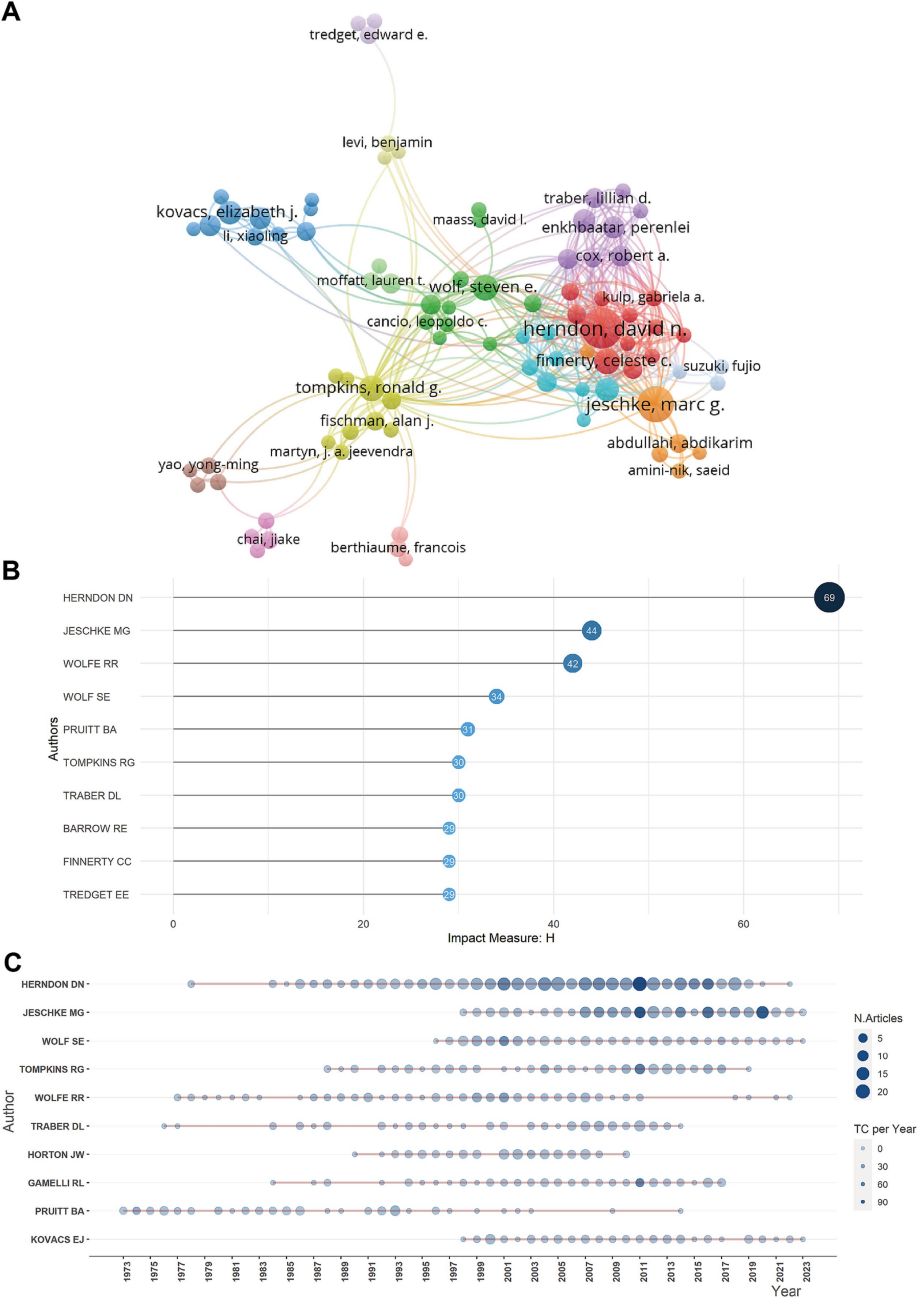
Authors analysis. **(A)** Network map showing cooperation among authors. **(B)** Top 10 authors with the most local impact measured by H index for metabolism and burns research. **(C)** Authors’ production over time for metabolism and burns research.

A total of 28,611 authors had contributed to the body of literature on burn metabolism (Figure 3A). Herndon DN, having authored a remarkable 358 publications, stood out as the most prolific author, followed closely by Jeschke MG with 160 publications (Supplementary Figure S4A). Both authors claim the highest number of citations (Supplementary Figure S4B) and H-index (Figure 3B), highlighting their significant influence in the field. The authors’ productions (Figure 3C) showed that Marc G. Jeschke, Wolf SE, and Kovacs EJ had been relatively active authors recently.

Supplementary Table S4 and Supplementary Figure S4C displayed the top 10 most locally cited documents in the field of burn metabolism. The high number of local citations for these articles signified their significant impact and influence within the research community. Of these 10 articles, seven had more than 300 global citations. Among them, Wilmore DW’s article titled “Catecholamines: Mediator of the Hypermetabolic Response to Thermal Injury” ranked first with 274 local citations. The article, published in the Annals of Surgery in 1974, demonstrated that the elevated metabolic rate in burn patients was mainly due to the effect of catecholamines (20). This noteworthy achievement highlighted the pivotal role of this article in shaping the field of burn metabolism.

### 3.4 Journal impact and core sources

**Figure 4:**
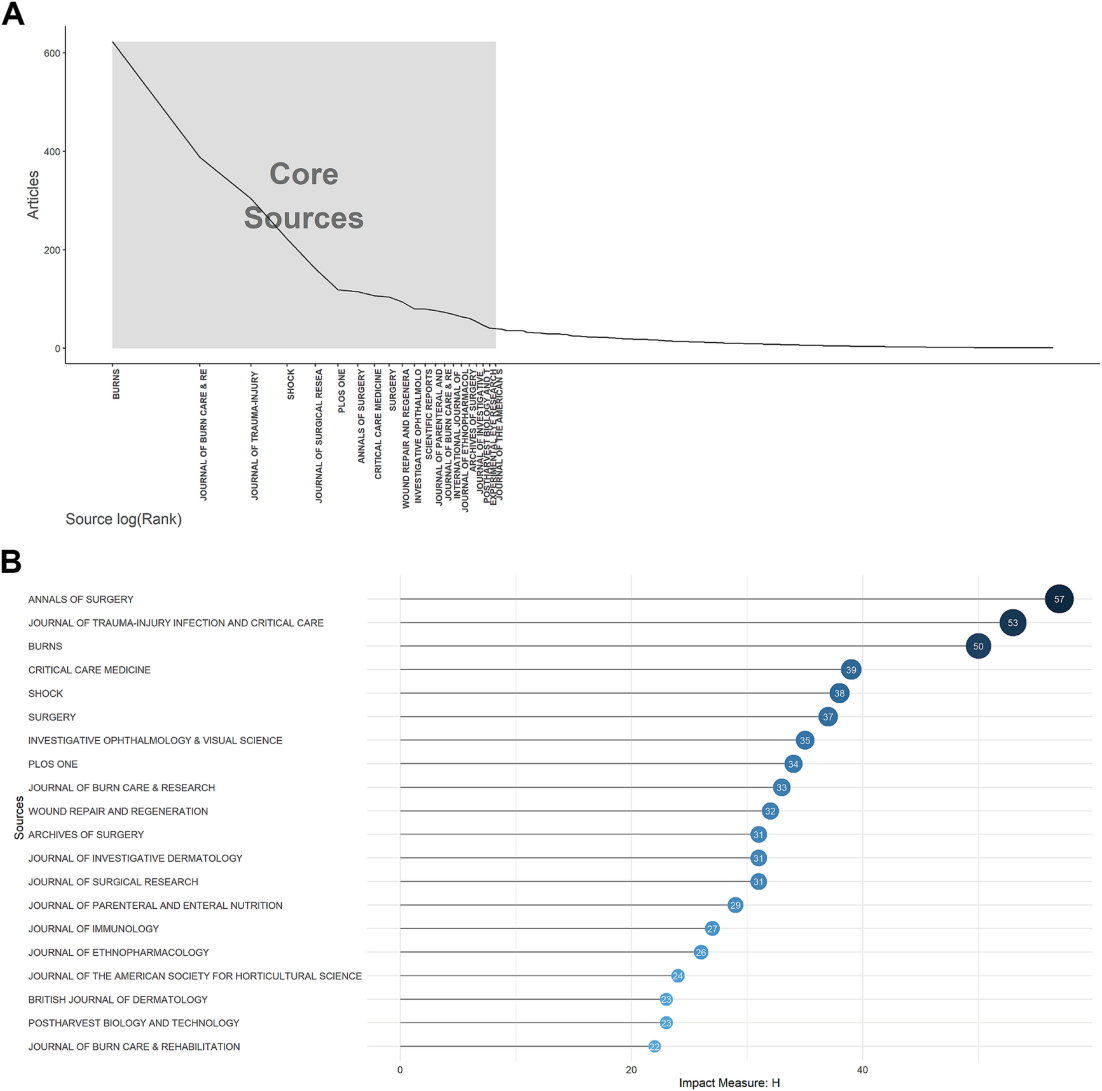
Journals analysis. **(A)** Core sources according to Bradford’s law for metabolism and burns research. **(B)** Top 20 journals with the highest local impact measured by H-index for metabolism and burns research.

As shown in Supplementary Figures S5A,B, the top 20 journals with the most documents and local citations. The analysis revealed that the preeminent journal in the field of burn metabolism was Burns, ranking first with 623 relevant publications and 10,057 citations. According to Bradford’s Law (Figure 4A), 21 journals were identified as core sources, based on their publication volume, while others exhibited a more evenly distributed literature output. Examining local impact, as depicted in Figure 4B, Annals of Surgery, Journal of Trauma-injury Infection and Critical Care, and Burns stood out as the top three journals, boasting H-indexes of 57, 53, and 50, respectively.

### 3.5 Keywords analysis

**Figure 5:**
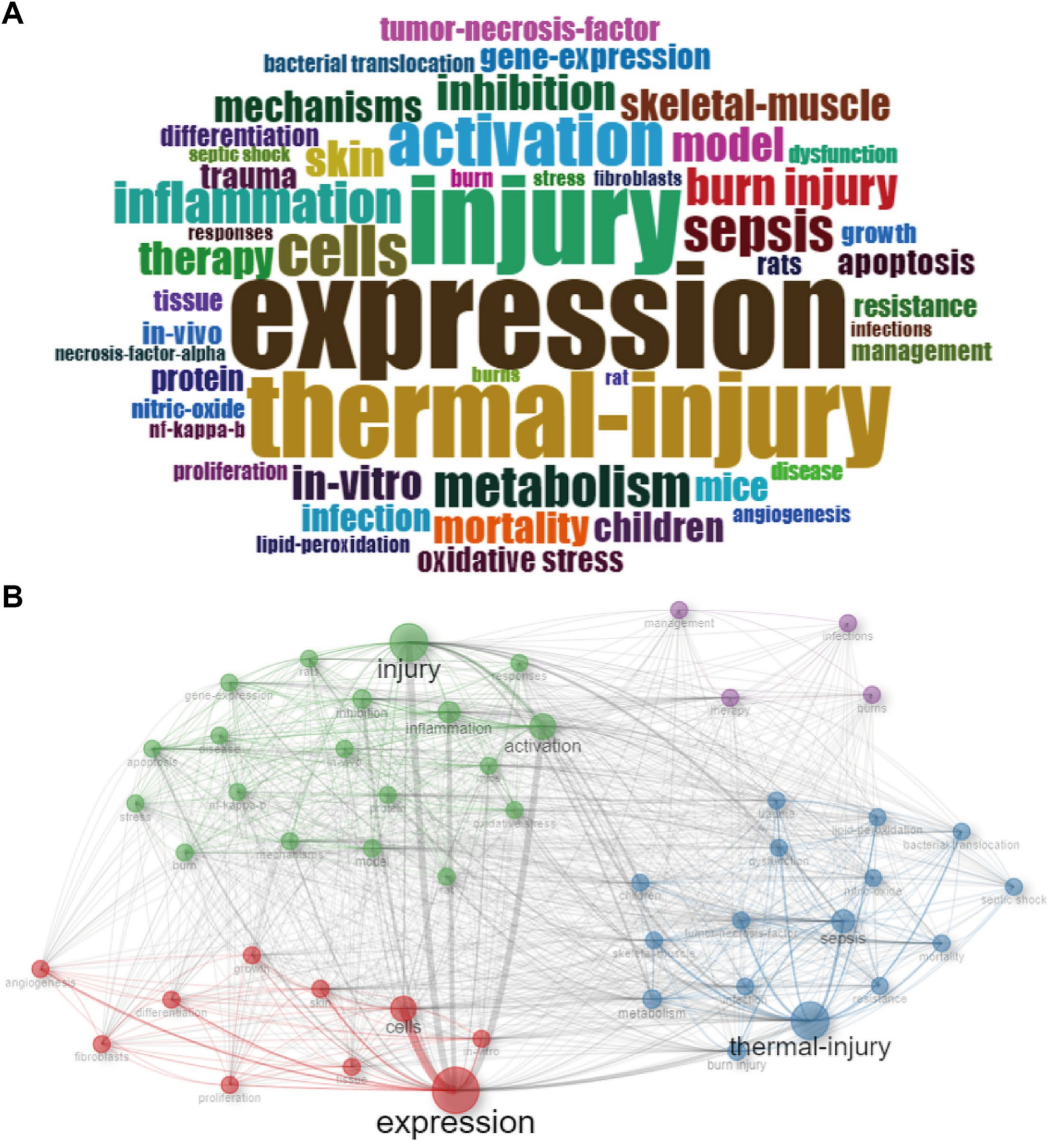
Keywords analysis. **(A)** Visualized word cloud based on the top 50 most frequent keywords for metabolism and burns research. **(B)** Co-occurrence network for metabolism and burns research.

Keywords serve as concentrations of critical research content within the literature. In our study, we meticulously examined the keywords extracted from 8,823 documents. The word cloud (Figure 5A) and tree map (Supplementary Figure S6A) visually portrayed the top 8 most frequent keywords, revealing their respective frequencies: “expression” (815), “injury” (677), “thermal-injury” (648), “activation” (402), “cells” (388), “sepsis” (334), “metabolism” (315), “inflammation” (314). Notably, Supplementary Figure S6B elucidated an overall increasing trend for these keywords, with “expression” exhibiting the highest frequency and the fastest growth rate. The trend topics (Supplementary Figure S6C) further illuminated the evolving landscape, showcasing the emergence of novel research keywords in recent years, such as “fabrication,” “dressings,” “antibacterial,” “antioxidant activity,” and “care.”

#### 3.5.1 Co-occurrence network

With the co-occurrence network (Figure 5B and Supplementary Figure S7), we linked related keywords and marked them with different colors. Keywords with the same color form a cluster. From this figure we can see 4 clusters:

Cluster 1 (red): The examination of gene expression variations across different cell types post-thermal skin injury. The crucial keywords include “expression,” “cells,” and “skin.”

Cluster 2 (blue): The metabolism shifts and sepsis onset following thermal injury. The crucial keywords include “thermal-injury,” “sepsis,” and “metabolism.”

Cluster 3 (green): The post-injury inflammation response activation. The crucial keywords include “injury,” “activation” and “inflammation.”

Cluster 4 (purple): The comprehensive management and therapeutic strategies for burn patients. The crucial keywords include “therapy,” “management” and “burns.”

#### 3.5.2 Thematic map

**Figure 6:**
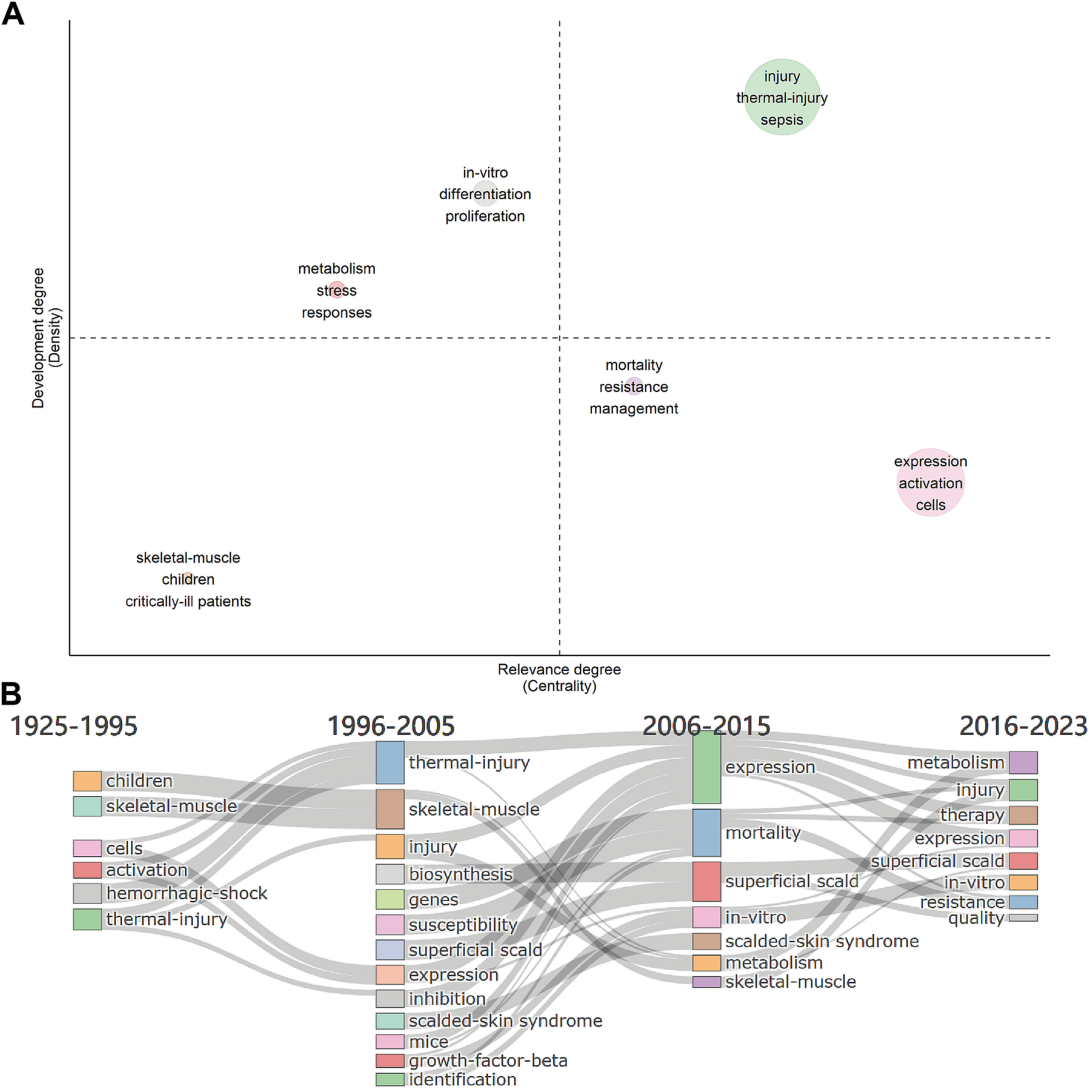
Keywords analysis. **(A)** Thematic map for metabolism and burns research. The density represents development, the centrality represents the importance. Upper right, upper left, lower left, and lower right are quadrants 1 to 4, representing motor themes, niche themes, emerging/declining themes, and basic themes, respectively. **(B)** Thematic evolution from 1925 to 2023, with cut-off points in 1995, 2005 and 2015 for metabolism and burns research.

In the thematic map, density was represented by the vertical axis, and centrality was represented by the horizontal axis, which together measure the development and importance of the research. The higher the density, the better the development; the higher the centrality, the more critical. In our research, the literature’s keywords could be categorized into six themes based on their connections (Figure 6A).

Motor themes are in the first quadrant, which represents the topic of high centrality and high density. In this quadrant, keywords were “injury,” “thermal-injury,” and “sepsis” (circle green), which meant the study of the pathogenesis of burn sepsis was essential in this field and was relatively mature. The topic of how the body responds to thermal injury (circle red) and *in-vitro* differentiation and proliferation of different cells (circle gray) were in the second quadrant with high density but low centrality, which meant they were well-developed but of lower importance. In the third quadrant was the pathological changes of skeletal muscle in critically ill patients and children (circle brown), which was an emerging/declining theme and needed to be expanded. There were two topics in the fourth quadrant. The topic of managing drug-resistant infections in burn patients (circle purple) had a low density, meaning that the research was underdeveloped. Infections were the leading cause of death in burn patients. However, the management and prevention of multiple drug resistance (MDR) infections in burn patients is still an ongoing challenge (21). The topic of expression of genes and activation of cells or pathways in cells after burn injury (circle pink) was of high centrality, indicating that it is the basis and focus of current relevant research.

#### 3.5.3 Thematic evolution

We selected 3 cutting points (in years), 1995, 2005, and 2015, according to the time span of the data. The thematic evolution (Figure 6B) showed how the main research themes had changed. In the early period (1925–1995), the themes included “children,” “hemorrhagic-shock,” “skeletal-muscle,” “activation,” “cells,” and “thermal-injury.” This implied that the study of pediatric thermal injury and the observation and symptomatic management of patients may have been the focus of research at that time. At the same time, cell expression studies were gradually developed. The themes of the second phase (1996–2005) evolved into “thermal-injury,” “skeleton-muscle,” “injury,” “expression,” “inhibition,” “superficial-scald,” and “critically-ill patients,” while some new topics such as “biosynthesis,” “genes,” etc. had emerged. It suggested that there were more studies on the cellular and molecular mechanisms of burns at that time. The themes of the third phase (2006–2015) evolved into “expression,” “mortality,” and “*in-vitro*,” etc., suggesting an increase in the number of *in-vitro* experiments. The themes of the latest phase (2016–2023) developed into “resistance,” “*in-vitro*,” and “therapy,” etc., suggesting that the drug-resistant infection and the therapy of hypermetabolism and disfunction of burn patients had become hot topics.

## 4 Discussion

Burns lead to systemic metabolic alteration and inflammatory responses, which greatly affect the prognosis of patients. Over the past several decades, researchers have demonstrated the relationship between inflammatory immune response and postburn hypermetabolism and evaluated the effects of drug and nutrient provision. In our investigation, we applied a data visualization approach to analyze a large number of articles within the domain of burn metabolism, totaling 8,823 retrieved articles. The paramount contributors in terms of countries, and authors were the United States and David N Herndon, respectively. The preeminent journals making a significant local impact on burn metabolism research were elucidated as Annals of Surgery, Journal of Trauma-injury Infection and Critical Care, and Burns. Additionally, our analysis points towards potential directions for future research endeavors. We conducted a thorough analysis of keywords to construct a co-occurrence network, revealing four pivotal research hotspots in the field of metabolism and burns. These include the examination of gene expression variations across different cell types post-thermal skin injury, the metabolism shifts and sepsis onset following thermal injury, the post-injury inflammation response activation, and the comprehensive management and therapeutic strategies for burn patients. Our thematic map underscored the significance of research into the management of drug-resistant infections as a critical and emerging area that warrants focused attention. In summarizing these research hotspots within the realm of burn metabolism, our study provides a comprehensive overview, delineating critical areas for exploration and fostering a deeper understanding of the evolving landscape within this pivotal domain of medical research. In the preceding Co-occurrence Network, we delineated four clusters. Based on content relevance, Cluster 1 (The examination of gene expression variations across different cell types post-thermal skin injury), Cluster 2 (The metabolism shifts and sepsis onset following thermal injury) and Cluster 3 (The post-injury inflammation response activation) are merged and discussed collectively under the umbrella of “Activation of Inflammation and Metabolic Changes in Burn Patients.” Cluster 2 (The metabolism shifts and sepsis onset following thermal injury) is also strategically combined with Cluster 4 (The comprehensive management and therapeutic strategies for burn patients), culminates in the formation of the section “Clinical Management: Pharmacotherapy, Nutritional Intervention, and Sepsis**.”**

### 4.1 Activation of inflammation and metabolic changes in burn patients (cluster 1, cluster 2, and cluster 3)

Burn injuries elicit a profound inflammatory response in patients, and the intensity of this response is directly correlated with the severity of the burn, with a larger total body surface area (TBSA) resulting in a more robust inflammatory response (22). Molecules such as catecholamines (20, 23), glucocorticoids (24), cytokines (25, 26), and acute-phase proteins (27, 28), etc. are elevated after burns, and the extent of these elevations correspond to the severity of the injury.

**Figure 7:**
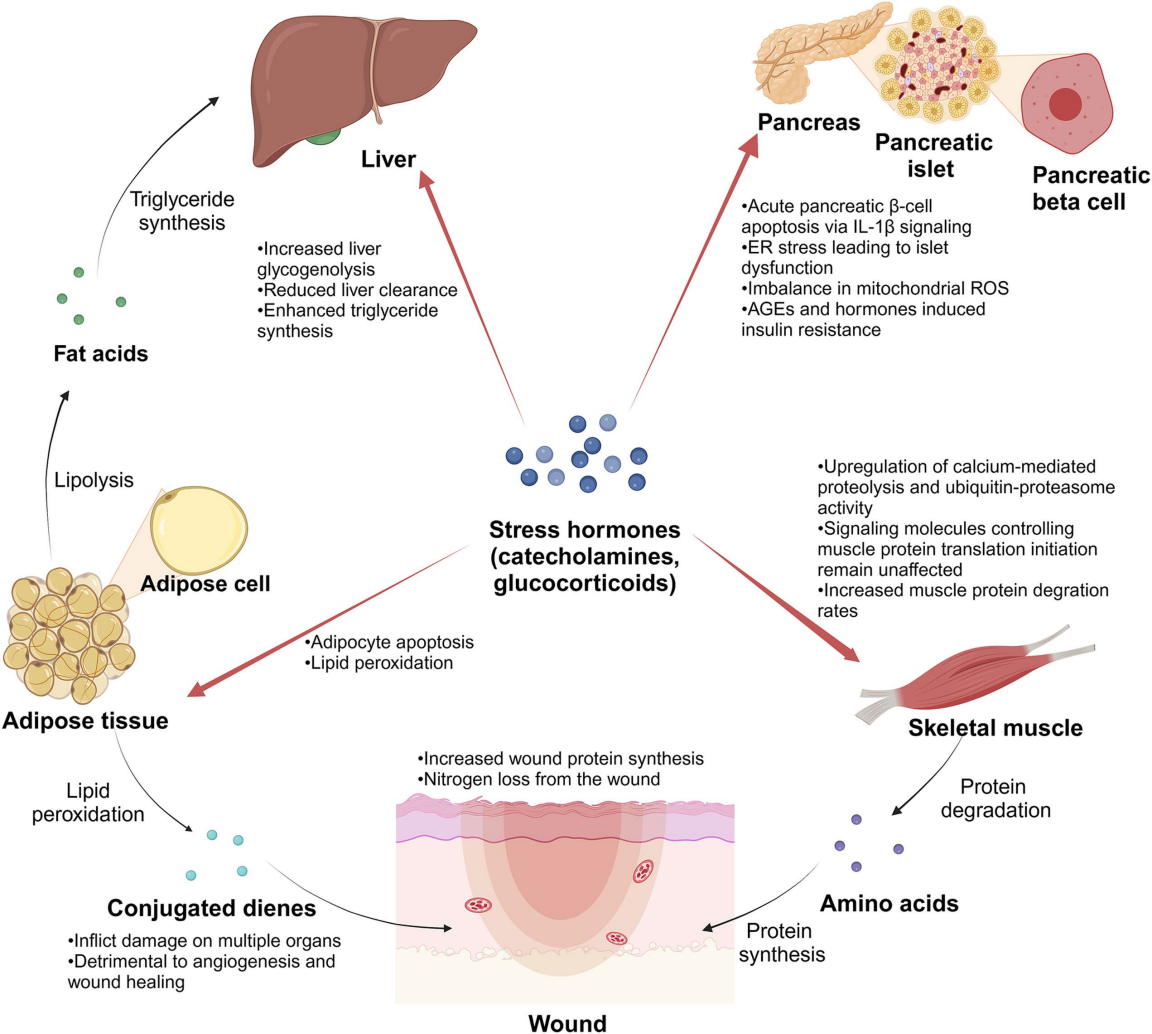
Schematic diagram. Stress hormones play an important role in the metabolic changes of burn patients after suffering severe burns. Created in BioRender (141).

Within this hyperinflammatory state, there is a concurrent escalation in the metabolic activity of burn patients. Jeschke et al. (29) focused on severely burned children, assessing parameters such as resting energy expenditure (REE), body composition, metabolic markers, and cardiac and organ function. The findings affirmed that burns induce metabolic hyperactivity within 3 years post-injury, accompanied by a heightened inflammatory state (29). Comparable conditions have also been observed in adults to varying degrees through studies conducted by diverse researchers (5, 30). The primary nutrients – glucose, fat, and amino acids – undergo oxidative decomposition in the body to supply energy. In the aftermath of extensive burn injuries, there is a transient suppression of energy consumption, plunging the body into a state of metabolic suppression (ebb phase) lasting 1–2 days, potentially extending to 3–4 days in critically ill patients, aligning with the shock phase (31). Subsequently, patients transition into a state of metabolic hyperactivity (flow phase), characterized by elevated glucose production, intensified lipolysis, robust protein catabolism, and a substantial surge in energy consumption (6). This metabolic state of hyperactivity may persist for several months or even years. Alterations in body status induced by metabolic factors, such as elevated plasma glucose (32), high plasma fatty acids (33), and hypoproteinemia (34), can significantly impact wound healing, overall recovery, prognosis, and the emergence of complications (Figure 7).

#### 4.1.1 Changes in glucose metabolism in burn patients

In the initial stages of the burn, burn patients frequently exhibit hyperglycemia resulting from altered glucose metabolism induced by stress, inflammation, and other factors. This hyperglycemic state, termed stress hyperglycemia, is primarily orchestrated by a complex interaction of counter-regulatory hormones such as catecholamines, growth hormone, cortisol, and cytokines (32). While stress hyperglycemia typically resolves after the acute phase, burn patients often continue to experience hyperglycemia (35). This persistence may be linked to compromised pancreatic islet structure and function, relative insulin synthesis and secretion deficiency after severe burns, and the impact of inflammatory and stress responses on insulin secretion in adult patients. Studies have unveiled specific mechanisms contributing to post-burn hyperglycemia, including IL-1*β*-induced pancreatic β-cell apoptosis (36), endoplasmic reticulum (ER) stress leading to islet dysfunction (37), and an imbalance in mitochondrial reactive oxygen species (ROS) production in islet cells (38). Additionally, insulin resistance induced by factors like advanced glycosylation end products (AGEs) (39) and hormones such as glucocorticoids further complicates the scenario (40, 41), influencing blood glucose utilization and enhancing gluconeogenesis from sources like lactate, protein, and fat. This intricate interplay underscores the multifaceted nature of hyperglycemia in the later stages of burn injury, potentially establishing a vicious cycle that warrants meticulous clinical attention and intervention.

#### 4.1.2 Changes in protein metabolism in burn patients

Following severe burns, patients exhibit pronounced muscle hyperproteolysis, and plasma concentrations of amino acids undergo dynamic changes. Merritt et al. (42) discovered elevated circulating levels of proinflammatory cytokines in burn patients, with concurrent upregulation of calcium-mediated proteolysis and ubiquitin-proteasome activity. However, signaling molecules controlling muscle protein translation initiation remain unaffected (42), which indicates that the net catabolic effect of burn injury in skeletal muscle may be mediated by increased proteolysis rather than decreased protein synthesis. Muscle protein degradation increases within 24 h after admission (43), and amino acids are released from the muscle, acting as an amino acid pool for wound repair (44). Within the first 6 h post burn, the plasma amino acid values in patients with body surface area (BSA) over 50% show a significant increase, including Alanine (Ala), hydroxyproline (Hyp), phenylalanine (Phe), and so on, while in the patients with BSA under 50% are not significantly different from usual (45). During the initial 7 days, concentrations of glucose precursor amino acids in patients (BSA over 20%) decrease, potentially linked to increased liver glucose synthesis and acute-phase protein synthesis (46). Simultaneously, plasma concentrations of Phe increase (46). The plasma concentrations of branched-chain amino acids, proline (Pro), and ornithine (Orn) remained unchanged until the 3rd week, then rose markedly (46). Notably, wound protein synthesis rates increase (47), indicating that the wound consumes amino acids to support healing. Protein is predominantly lost as urea and exudates from open wounds in the first week post-injury (48). Ultimately, the enhanced protein degradation and decreased protein synthesis metabolism generally result in a negative nitrogen balance, accompanied by symptoms of notable muscle weakness and atrophy.

#### 4.1.3 Changes in lipid metabolism in burn patients

Severe burns trigger inflammatory reactions, intensifying lipid metabolism. Blood lipid, triglyceride, and free fatty acid levels are significantly elevated throughout acute hospitalization (33). In patients with burns, the beta 2-receptor for catecholamines triggers lipolysis (49). Beta-adrenergic stimulation primarily stimulates triglyceride-fatty acid-substrate cycling (50). Interestingly, some burn patients may exhibit desensitization of the signal transduction pathway downstream of beta-adrenergic receptors (51). In addition, elevated plasma concentrations of lipid metabolic byproducts persist, potentially linked to adipocyte apoptosis (52) and reduced liver clearance of metabolites (51). Burn injuries also prompt fat redistribution, with increased muscle utilization of fat, enhanced triglyceride synthesis from fatty acids in the liver, and notable adipocyte infiltration (53). Burn injury decreased epidermal lipid production and lipid synthesis enzymes in donor skin and burn margin (54). This disruption of the epidermal barrier function can have detrimental effects on crucial processes such as wound healing and graft survival (54). Furthermore, burns trigger oxidative activation in the body, leading to lipid peroxidation. This process generates various lipid oxidation products, including conjugated dienes, that can damage multiple organs (55). In addition, Altavilla et al. have demonstrated that inhibiting lipid peroxidation can effectively enhance angiogenesis and promote wound healing (56).

### 4.2 Clinical management: pharmacotherapy, nutritional intervention, and sepsis (cluster 2 and cluster 4)

**Figure 8:**
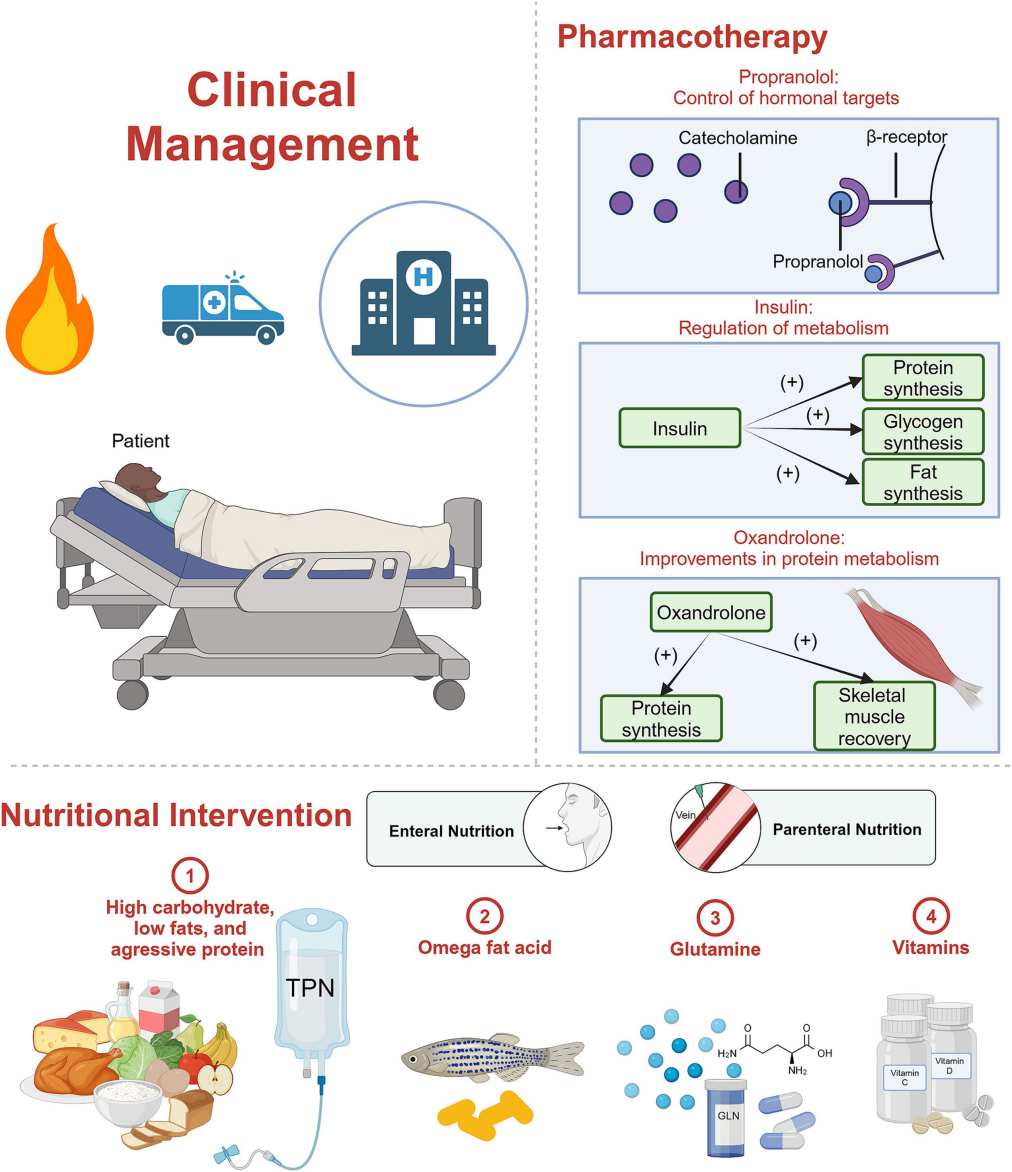
Schematic diagram. Clinical management for burn patients, including pharmacotherapy and nutritional intervention. Created in BioRender (142).

Severe burns trigger a cascade of metabolic changes that persist over time, significantly affecting the patient health. Addressing these metabolic alterations necessitates both medication therapy and nutritional support, as illustrated in Figure 8. The compromised skin barrier post-burn not only fails to protect against external threats but also becomes a breeding ground for infections, such as sepsis, which can further disrupt metabolic processes. Consequently, clinical infection control is crucial for preventing the escalation of these metabolic disorders and promoting recovery.

#### 4.2.1 Medication therapy

The elevated metabolic levels observed in burn patients are closely linked to the inflammatory response. The objective of medication therapy targeting hypermetabolism is to reduce the effect of the inflammatory response and to manage a spectrum of associated metabolic disorders.

A repertoire of drugs has undergone extensive study for their efficacy in addressing hypermetabolism in burn patients (57). Prominent among these are propranolol, insulin, and oxandrolone. Insulin and oxandrolone are anabolic agents that regulate blood glucose and protein metabolism. Propranolol, an anti-catabolic agent, is used to block the beta-adrenergic receptor, which was proven to be associated with the increased catabolic rate (20). Additionally, medications such as recombinant human growth hormone (rHGH) (58), IGF-1 (59), Insulin Growth Factor Binding Protein 3 (IGFBP-3) (59), and metformin (60) have demonstrated potential in alleviating the hypermetabolic response in burn patients. These pharmacological interventions stand as crucial avenues in the pursuit of effective strategies to manage hypermetabolism, emphasizing the ongoing need for rigorous research to refine treatment modalities for individuals afflicted by severe burn injuries.

##### 4.2.1.1 Propranolol: control of hormonal targets

Propranolol, a non-selective beta-blocker, functions by impeding the binding of endogenous catecholamines to beta receptors. This pharmacological action reduces heart rate, weakened myocardial contraction, and diminished cardiac output.

Herndon et al. highlight the efficacy of propranolol in pediatric burn patients, showcasing its ability to decrease myocardial workload (61) significantly. Administering propranolol over 1 year has demonstrated improvements in hemodynamics, reduced REE, and mitigated central adipose deposition (62). Sylvia Ojeda et al.’s study involving 104 burned children affirms the safe use of propranolol to treat hypermetabolic-related tachycardia in children post-burn injury, with no adverse effects observed upon immediate discontinuation (63). Moreover, the combination of propranolol with rHGH exhibits a synergistic effect, significantly reducing hypermetabolism and inflammation (64). Integrating beta-blockers during rehabilitation exercise training (RET) in children has demonstrated enhanced physical function during rehabilitation (65).

In the context of adult burn patients, propranolol yields similar positive effects but is associated with an increased incidence of adverse reactions. Sarah Rehou et al. revealed varied outcomes, with benefits such as improved insulin sensitivity, decreased peripheral lipolysis, reduced inflammatory mediators, normalized metabolic profiles, and improved prognosis (66). Propranolol’s multifaceted impact extends to reducing bleeding, promoting wound healing (61), influencing the inhibitory effect of M2 macrophages on M1 macrophages (67), and limiting hepatic fat storage by impeding fatty acid transport (68). However, a study underscores that while effective, propranolol does not significantly reduce mortality or the incidence of sepsis in critically ill burn patients (69). Moreover, Brown et al. (70) found that in adult burn patients using propranolol (mean dosage was 0.46 mg/kg/day), 72% experienced at least one episode of hypotension, 15% experienced bradycardia, and there was a high incidence of hold events. The impact of propranolol on prolonging mechanical ventilation duration has been confirmed in ventilator-dependent surgical patients (71).

##### 4.2.1.2 Insulin: regulation of blood glucose

Severe burn injuries induce profound metabolic disturbances, prominently marked by heightened catabolism leading to hyperglycemia in most cases. Insulin, a potent synthetic metabolic agent, proves effective in mitigating elevated blood glucose levels in burn patients, albeit with intricacies surrounding its optimal dosage and mechanism of action.

Insulin exhibits a multifaceted role in modulating inflammatory responses. The research underscores its capacity to balance inflammatory factors, elevate specific anti-inflammatory cytokine expression, and reduce pro-inflammatory factors (72, 73). In a dose-dependent manner, insulin demonstrates an anti-inflammatory effect, notably decreasing plasma levels of sTREM-1, TNF-*α*, IL-6, and HMGB1 in severely scalded rats with multiple organ dysfunction syndrome (MODS) (74). Additionally, low-dose insulin treatment enhances resistance to *Pseudomonas aeruginosa* burn wound infection, potentially due to improved inflammatory and immune responses (75).

Insulin exerts protective effects on various organs. It enhances liver structure and integrity by promoting hepatocyte proliferation and reducing apoptosis, as evidenced by decreased caspase-3 activation and increased Bcl-2 expression in burn rats (72, 76). Insulin therapy contributes to primary myogenesis, reducing muscle atrophy, improving muscle function, and synergizing positively with exercise training (77, 78). Wound healing quality and collagen deposition are also augmented in rats treated with insulin, likely linked to improved protein metabolism and amino acid redistribution (77, 79).

Intensive insulin therapy (IIT), maintaining blood glucose levels within a specific range, offers benefits such as improved mitochondrial function (80), enhanced insulin sensitivity, and reduced infection/sepsis incidence (81, 82). The application, however, poses challenges such as an increased risk of hypoglycemic events and the necessity for patients to consume a substantial amount of exogenous glucose to prevent hypoglycemia (83). Ferrando et al. (84) indicate varying insulin doses affect net protein synthesis in skeletal muscle without compromising glucose uptake.

##### 4.2.1.3 Oxandrolone: improvements in protein metabolism

Oxandrolone, a synthetic anabolic androgenic steroid (AAS) derived from dihydrotestosterone, emerges as a valuable intervention in the context of burn-induced metabolic response and associated complications. Notably, it exhibits muscle-enhancing properties with reduced masculinizing activity compared to testosterone.

In burn patients, the heightened metabolic response triggers substantial protein catabolism, muscle wasting, and a decline in lean body mass (LBM) (85). Oxandrolone proves beneficial by effectively ameliorating the inflammatory response (86, 87), enhancing protein metabolism, and correcting negative nitrogen balance (88). It expedites skeletal muscle recovery, leading to significantly shortened hospital stays (89). For burned children experiencing growth issues, oxandrolone aids in improving bone mineral content (BMC) (90, 91). Combining oxandrolone with propranolol accelerates skeletal muscle recovery, shortens growth arrest duration (92, 93), and reduces post-burn hypertrophic scarring (94).

Clinical trials reveal that pediatric patients on 0.1 mg/kg oxandrolone twice daily experience increased muscle protein synthesis without altering protein degradation (95, 96), potentially attributed to enhanced amino acid utilization (97). Long-term use (1 year) at this dosage significantly boosts serum IGF-1, T3 uptake, and FTI in children without adverse effects, indicating the safety of oxandrolone (91, 98). Oxandrolone’s influence on gene expression, particularly downregulating stress-related genes and upregulating those associated with muscle hypertrophy and oxidative damage modulation, underscores its positive impact in burn children (88, 99). Similar positive effects extend to adult burn patients (100, 101).

However, caution is warranted, as a 10 mg oxandrolone dosage twice daily may elevate transaminase levels in patients, with identified risk factors including younger age and concurrent use of intravenous vasopressors and amiodarone (102). Although liver enzymes aspartate aminotransferase (AST) and alanine aminotransferase (ALT) increased in the short term, serum levels of IGF-I, IGFBP-3, and growth hormone remained unaffected, suggesting that oxandrolone may not have hepatotoxicity (95). However, its effect on burn patients remains unverified, thus vigilance in oxandrolone usage is essential, considering potential implications.

#### 4.2.2 Nutritional intervention

Burn patients experience a hypermetabolic response in the flow phase, resulting in significant energy loss. At this stage, providing essential nutritional support to burn patients is aimed at meeting their basic nutritional needs and preventing poor nutritional status caused by the hypermetabolic state, which can adversely affect patients’ recovery. Therefore, supplementing appropriate amounts of nutrients and compensating for the body’s energy expenditure is beneficial for the rehabilitation of burn patients.

##### 4.2.2.1 Nutritional support modalities

Enteral nutrition (EN) and parenteral nutrition (PN) are two main nutritional support methods, each of which has its advantages and considerations. PN provides reliable bioavailability and allows for rapid administration compared to EN (103, 104). However, Researchers have suggested that total parenteral nutrition (TPN) may have drawbacks, including increased TNF-*α* expression and production, reduced survival rates in thermal injury rats, heightened endotoxin translocation, intestinal atrophy, and an elevated stress response (105, 106). Conversely, early EN in burn patients has demonstrated various benefits. It increases levels of serum gastric and plasma motilin, alleviates gastrointestinal damage, preserves intestinal mucosal integrity after severe burns, and mitigates ischemia–reperfusion injury by counteracting oxidative stress (107–109).

While most studies advocate for the advantages of early EN (110), challenges persist, particularly in the early stage of severe burn cases (111). Obstacles such as paralytic ileus, peritonitis, upper gastrointestinal hemorrhage, intractable vomiting, and severe diarrhea can impede EN delivery. In such scenarios, PN serves as a vital alternative nutrition source. Consequently, a consensus emerges from existing literature recommending EN as the primary approach, supplemented by judicious use of PN when necessary (103, 112). Supplemental parenteral nutrition (SPN) appears as a strategic solution, accurately achieving energy goals, improving energy supply, reducing antibiotic usage, and lowering the risk of infection (113). Additionally, SPN exhibits significant enhancements in immune function (114).

##### 4.2.2.2 Comprehensive nutrient considerations

Burn patients undergoing a hypermetabolic response necessitate targeted nutrient supplementation to address nutrient decomposition and severe energy expenditure. However, both malnutrition and overnutrition adversely affect burn patient outcomes. Therefore, it’s crucial to monitor energy expenditure changes and select an appropriate nutrition regimen.

Supplement of high-quality carbohydrates, fats, and proteins is needed. Shields et al. suggested high-carbohydrate, low-fat diet is proposed to enhance insulin and insulin-like growth factor levels, improving metabolism (115). Alexander et al. (116) suggested that aggressive protein feeding proves beneficial, with regular diets often insufficient, emphasizing the importance of high-protein supplementation.

Omega fatty acids have anti-inflammatory and anti-oxidative effects (117). Adding omega-3 polyunsaturated fatty acids to the low-fat diet has shown clinical benefits, reducing severe sepsis, septic shock, and pyloric dysfunction (118). When used with collagen, omega-3 fatty acids reduce high-sensitivity C-reactive protein (119). However, Siritientong et al. (120) found no benefits of omega-3 fatty acids in lowering various complications, mortality, and hospital stays in burn patients. Thus, the clinical benefits of omega-3 fatty acids still need to be validated.

Glutamine, a free *α*-amino acid, has been linked to improved metabolism and inflammatory stress responses (121). Some researchers have shown that it can improve nitrogen balance and protect organs (122), and enteral glutamine for treatment may also enhance intestinal permeability (123). In a meta-analysis, glutamine supplementation was significantly associated with a reduction in mortality rates and hospital stays (124). However, a recent large-scale randomized controlled trial did not show improvement in burn patients’ hospitalization length (121). In conclusion, the application of glutamine in burn patients remains controversial.

Vitamins are micronutrients that are essential to our body. A retrospective cohort study showed that vitamins and minerals demonstrated overall benefits in burn patient care (125). Presently, there is more research on vitamin D in the field of burns. Apart from its role in maintaining calcium and phosphorus metabolism, it also preserves immunity (126). Vitamin D deficiency is prevalent in burn patients, impacting wound healing time, increasing sepsis incidence, and prolonging hospital stay (127–129). However, how vitamin D affects the prognosis and metabolism of burn patients still needs to be clarified. Ascorbic acid (vitamin C) mitigates oxidative stress damage, reduces systemic inflammation, and influences fluid demand post-burn (130–132).

#### 4.2.3 Sepsis and drug-resistance in burn care

Burns compromise the skin’s protective barrier and alter intestinal permeability, creating an environment conducive to bacterial invasion and a heightened risk of sepsis. Schwacha et al. (133) have elucidated the pivotal role of macrophage-activated proinflammatory cascades post-thermal injury in the progression of immunosuppression and the increased vulnerability to sepsis in burn patients. This immunological upheaval is further exacerbated by the acute insulin resistance that often accompanies burns, intensifying hypermetabolism, disrupting glucose metabolism, and precipitating immune dysregulation, thereby elevating the risks of sepsis (134). Sepsis, in turn, instigates a cascade of systemic metabolic and inflammatory disturbances, culminating in escalated energy demands that drive the catabolism of both adipose and lean tissues (85). Diao et al. (135) confirmed that the combination of burn injury and infection stimulates lipolysis in white adipose tissue, in part through the induction of ER stress, further contributing to hyperlipidemia and significant hepatic lipid deposition.

Moreover, the emergence of multidrug-resistant organisms among burn patients poses a formidable and ongoing challenge in the management and prevention of infections (21, 136). Infectious such as candidemia (137) and *Pseudomonas aeruginosa* infection (138) are not only leading causes of mortality and impediments to recovery in burn patients but also have a tendency to rapidly progress to sepsis.

Consequently, it is imperative to implement strategies that control infections, combat drug-resistant bacteria, and prevent the onset of sepsis. Despite the considerable advancements in medical care, the growing issue of drug resistance and its complex implications necessitate rigorous and ongoing research. There is an urgent need for further investigation to refine therapeutic approaches for managing hypermetabolism and associated dysfunctions in burn patients. The aim is to preempt the detrimental effects of metabolic complications and to enhance patient outcomes. In this context, the work of Anne Argenta and colleagues highlights the potential of local probiotic therapy as a valuable adjunct in the management of complicated burn injuries (139), offering a promising avenue for further exploration and clinical application. Additionally, ongoing research is focused on developing new antibiotics that are effective against resistant bacteria (140), providing hope for more effective treatment options in the future.

### 4.3 Limitations

While our study provides a comprehensive overview of burns and metabolism research, certain limitations merit consideration. The search, conducted on 10 July 2023, may omit publications postdating this period due to database updates. Additionally, the use of biblioshiny and VOSviewer for bibliometric analysis, lacking the capability to organize keywords, introduces potential biases. For example, if there is no way to identify and merge the keywords with the same meaning, then even if the research is relatively popular, it will not be reflected in the keyword analysis data, which may lead to a decrease in the popularity of research in this field in terms of data. Besides, although some articles were not related to our research in the field of burn metabolism, the keywords were the same. Such articles, if included in our database, may lead to them being listed as one of the research topics in this field. Moreover, some recent articles may be less cited than earlier ones because of their late publication, but they may become highly cited in the future. However, the analysis based on citation may lead to the omission of these recent, high-quality studies. To counteract these limitations, an extensive literature review and thematic trend discussions based on keywords were conducted, enhancing the robustness of our analysis. Furthermore, our analysis has inherent limitations. Some keywords were found to be overly broad, obscuring the specificity needed to convey detailed research themes effectively. Additionally, our affiliation-based analysis did not account for institutions that may be part of larger systems, which could impact the accuracy of our results. This limitation highlights the need for more sophisticated analytic models. These methodological considerations are yet to be fully addressed and will be considered in the ongoing refinement of our research methodologies.

## 5 Conclusion

In our investigation, we applied a data visualization approach to analyze a large number of articles within the domain of burn metabolism, totaling 8,823 retrieved articles. We analyzed the countries, affiliations, journals, and authors. We find that the most contributed countries and authors were the United States, and David N Herndon, respectively. The most influential journals were Annals of Surgery, Journal of Trauma-injury Infection and Critical Care, and Burns. We found an upward trend in the study of burns and metabolism. We analyzed keywords and found four pivotal research hotspots in metabolism and burns research, including changes in the expression of genes of different cells after skin thermal injury, metabolism change and incidence of sepsis after thermal injury, activation of inflammation after injury, and management and therapy for burn patients. In addition, addressing the challenges of drug resistance and sepsis are emerging as important areas for focused attention.

## Data Availability

The original contributions presented in the study are included in the article/Supplementary material, further inquiries can be directed to the corresponding authors.
