# Supplementary material for: Metabolic response to burn injury: a comprehensive bibliometric study
Source: Front Med (Lausanne). 2025 Jan 3;11:1451371. doi: 10.3389/fmed.2024.1451371 (PMC11739346; doi:10.3389/fmed.2024.1451371)
Supplement: Supplementary file 1 [file Table_1.docx]

**CO-EDITORS-IN-CHIEF**

**Michel Goldman**

**Institut pour l'Innovation Interdisciplinaire en Santé, Université Libre de Bruxelles, Brussels, Belgium**

**Frontiers in Medicine**

**Dear editors:**

We have conducted a clinical bibliometric analysis, entitled ‘**Metabolic Response to Burns: A Comprehensive Bibliometric Study**’, which we sincerely hope to be considered for publication in **Frontiers in Medicine**. On behalf of my colleagues, I would like to submit this manuscript of original research to **Frontiers in Medicine** and the manuscript had been read and approved for submission by all authors. All persons listed as authors had contributed to preparing the manuscript, and International Committee of Medical Journal Editors (ICMJE) criteria for authorship had been met and no person other than the authors listed had contributed significantly to its preparation.

Burns lead to systemic changes that are manifested by systemic disturbances in water-electrolyte balance and systemic metabolic and inflammatory responses. The hypermetabolic response after a thermal injury is based on metabolic, hormonal, and inflammatory dysregulation mechanisms. This study aimed to summarize the research hotspots in burn metabolism and evaluate global research trends through bibliometric analysis. In this work, scientific publications on metabolism and burns were obtained from the core collection of the Web of Science database, biblioshiny was used to visualize and analyze the data and VOSviewer was used to verify the results. From a total of 8,823 publications, we found a general upward trend in annual publications and citation frequency. According to Bradford’s Law, 21 high-production journals were classified as core sources based on the number of publications, and the most productive journal was Burns. The most published countries, institutions, and authors in this field were the United States, the University of Texas System, and Herndon DN. The most local cited document in this field was the article titled "Catecholamines: Mediator of the Hypermetabolic Response to Thermal Injury" authored by Wilmore DW. The thematic map showed that studies on injury, thermal injury, and sepsis were relatively mature. In contrast, research on metabolism, stress, and responses, and research on mortality, resistance, and management were less well-developed but were essential for the field. Based on the bibliometric analysis, we summarized two main themes: inflammation induces metabolic changes in burn patients and nutritional support. These topics represent current research hotspots.

It is noteworthy that the conclusions of our study are very meaningful for estimating the metabolotrophic state of the patients with burn injury and guiding the treatment of the patients, which was consistent with the purpose of **Frontiers in Medicine** that aimed to promote continued developments in surgery through the sharing of knowledge, ideas and good practice across all surgical specialties.

The contents of this manuscript are our original work and have not been published or under consideration for publication elsewhere, in whole or in part, prior to our submission of the manuscript to **Frontiers in Medicine**.

This study was supported in part by the National Natural Science Foundation of China (81930057, 81971836), CAMS Innovation Fund for Medical Sciences (2019-I2M-5-076), Clinical Key Discipline Project of Shanghai; Shanghai Top Priority Research Center Project (2023ZZ02013); the Excellent Academic Leader Project of Shanghai Science and Technology Committee (23XD1425000); Shanghai Rising-Star Program (Sailing Special Program) (No. 23YF1458400); the Postdoctoral Fellowship Program of CPSF (96926). The funders had no role in study design, data collection and analysis, decision to publish, or preparation of the manuscript.

We are very grateful for your time spent on reviewing this submission. We are looking forward to receiving comments from you. If you have any question regarding this submission or during the review process, please feel free to contact us at the address below.

(First correspondence author)

Shizhao Ji, MD, Department of Burn Surgery, the First Affiliated Hospital of Naval Medical University, Shanghai, People’s Republic of China (Email: shizhaoji2022@163.com)

Runzhi Huang, MD, Department of Burn Surgery, the First Affiliated Hospital of Naval Medical University, Shanghai, People’s Republic of China (Email: runzhihuang2022@163.com)

Dayuan Xu, MD, Department of Burn Surgery, the First Affiliated Hospital of Naval Medical University, Shanghai, People’s Republic of China (Email: dxdy2014@163.com)

2024/06/19
